# Associations between Healthcare Resources and Healthy Life Expectancy: A Descriptive Study across Secondary Medical Areas in Japan

**DOI:** 10.3390/ijerph17176301

**Published:** 2020-08-29

**Authors:** Rikuya Hosokawa, Toshiyuki Ojima, Tomoya Myojin, Jun Aida, Katsunori Kondo, Naoki Kondo

**Affiliations:** 1 Department of Human Health Sciences, Graduate School of Medicine, Kyoto University, Kyoto 606-8507, Japan; 2 Department of Community Health and Preventive Medicine, Hamamatsu University School of Medicine, Shizuoka 431-3192, Japan; ojima@hama-med.ac.jp; 3 Department of Public Health, Health Management and Policy, Nara Medical University, Nara 634-8521, Japan; motoya1014@gmail.com; 4 Department of Oral Health Promotion, Graduate School of Medical and Dental Sciences, Tokyo Medical and Dental University, Tokyo 113-8549, Japan; junaida916@gmail.com; 5 Division for Regional Community Development, Liaison Center for Innovative Dentistry, Graduate School of Dentistry, Tohoku University, Miyagi 980-8575, Japan; 6 Center for Preventive Medical Sciences, Chiba University, Chiba 263-8522, Japan; kkondo@kkondo.net; 7 Center for Well-being and Society, Nihon Fukushi University, Aichi 470-3295, Japan; 8 Center for Gerontology and Social Science, National Center for Geriatrics and Gerontology, Aichi 474-8511, Japan; 9 Department of Health and Social Behavior, School of Public Health, The University of Tokyo, Tokyo 113-0033, Japan; nkondo@m.u-tokyo.ac.jp; 10 Department of Health Education and Health Sociology, School of Public Health, The University of Tokyo, Tokyo 113-0033, Japan

**Keywords:** life expectancy, healthy life expectancy, Sullivan method, healthcare resources, secondary medical area, Japan

## Abstract

Japan has the highest life expectancy in the world. However, this does not guarantee an improved quality of life. There is a gap between life expectancy and healthy life expectancy. This study aimed to reveal the features of healthy life expectancy across all secondary medical areas (*n* = 344) in Japan and examine the relationship among healthcare resources, life expectancy, and healthy life expectancy at birth. Data were collected from Japan’s population registry and long-term insurance records. Differences in healthy life expectancy by gender were calculated using the Sullivan method. Maps of healthy life expectancy were drawn up. Descriptive statistics and correlation analysis were used for analysis. The findings revealed significant regional disparities. The number of doctors and therapists, support clinics for home healthcare facilities and home-visit treatments, and dentistry expenditure per capita were positively correlated with life expectancy and healthy life expectancy (correlation coefficients > 0.2). They also revealed gender differences. Despite controlling for population density, inequalities in healthy life expectancy were observed, highlighting the need to promote social policies to reduce regional disparities. Japanese policymakers should consider optimal levels of health resources to improve life expectancy and healthy life expectancy. The geographical distribution of healthcare resources should also be reconstituted.

## 1. Introduction

Although life expectancy (LE) is an indicator of health status, there is a growing interest in the quality of life (QOL) of older adults during their later years. Healthy life expectancy (HLE) is a useful indicator of a population’s overall health, reflecting length of life as well as QOL [1,2,3]. HLE refers to an individual’s length of life lived without limitations in daily activities. In addition, HLE at birth is an important indicator of a population’s health status and QOL [4]. Moreover, HLE combines data on both mortality and morbidity [5]. It summarizes mortality and non-fatal outcomes in a single measure of the general population’s health. 

The increasing age of the global population warrants greater attention to disorders. Although both LE and HLE have improved, LE with disability has also increased. In an aging society, as greater age puts increased pressure on social systems, extending HLE and shortening LE with disability are becoming global priorities [6]. Moreover, LE and HLE differ; longer LE increases the risk for disability [7,8]. Meanwhile, HLE increases more slowly than LE [9]. Efforts to shorten this gap and encourage healthy aging are needed. Moreover, HLE has been used to compare the health status of various populations and explore health inequalities within a given population. Inequalities in LE and HLE can be observed across multiple regions and countries. However, little is known regarding the determinants of inequalities in HLE at the regional level.

According to the World Health Organization (WHO), Japan had the highest average estimated LE at birth in 2016 with 84.2 years [10]. The LE of females in Japan was ranked first in the world with 87.2 years, while the LE of men was ranked sixth with 81.1 years. Similarly, Japan had the highest average HLE in 2016 with 74.8 years. However, the factors attributed to Japan’s longevity have been a source of debate. It is difficult to identify a specific factor since there are possibilities of interplay among various factors, such as healthcare system and lifestyle. According to studies, some of the possible factors include the country’s high living standards, medical advances, and the universal and accessible healthcare system [11,12]. 

Japan has achieved satisfactory population health at a reasonably low cost; in fact, the Japanese have universal health coverage with their National Health Insurance system. Although Japan has the highest levels of LE and HLE among the members of the Organisation for Economic Co-operation and Development (OECD), its healthcare expenditure as a share of Gross Domestic Product is below that of most OECD countries [13]. In addition, regarding lifestyle, the Japanese diet has a relatively lower calorie and fat intake compared to that of developed economies, such as Europe and North America. Several studies suggest that adopting a Japanese diet tends to reduce the risk of cardiovascular disease and metabolic syndrome [14,15]. 

However, on average, the Japanese spend the last 9.4 years (11.2% of LE) of their lives with poor health, mobility impairments, or in a bedridden state. The duration of the difference between LE and HLE represents the average number of years with poor health (i.e., unhealthy life expectancy) [16]. In Japan, there is still a gap between LE and HLE [17]. Although the Japanese have the highest LE and HLE in the world, unhealthy life expectancy is not necessarily at the highest levels (e.g., Singapore: 6.8 years; 8.2% of LE, Spain: 9.3 years; 11.2% of LE, Switzerland: 9.8 years; 11.8% of LE) [10]. In addition, having high LE does not necessarily mean high QOL. Thus, in recent years, the Japanese government has focused on extending HLE. 

Extension of HLE was one of the main goals presented in “The Second Term of the National Health Promotion Movement in the Twenty First Century” (Health Japan 21, the Second Term) [18,19]. This 10-year nationwide health promotion project from 2013 to fiscal year 2022 was developed by the Ministry of Health, Labour, and Welfare with two main goals: extending HLE and reducing health inequality. Health inequality refers to the gap in HLE among prefectures (Japan is divided into 47 administrative districts known as prefectures). A 2016 study calculated the HLE for each prefecture using data from the Comprehensive Survey of Living Conditions and reported a difference of 2.70 years for females and 2.00 years for males [20]. Although Japan has been successful in their health outcome statuses (e.g., reducing mortality and disability), variations in health outcome statuses between prefectures are increasing [21]. In fact, the government is considering setting a target health expenditure level for each prefecture to address the issue of rapid increase and regional variations in health expenditure. Although small municipal area analyses were conducted, the standard errors were too large. Furthermore, the population of several municipal areas is too small to provide medical care. Thus, to implement effective health policies, further descriptive analyses in smaller areas are required. It is important to clarify the difference in HLE in each healthcare accessible area and implement effective policies. 

In Japan, healthcare service areas have been established to provide efficient medical services. Under the Medical Care Law, these areas are required to provide general healthcare supplies through their prefectural governments [22,23]. Primary medical service areas consist of approximately 1700 districts; secondary medical areas consist of 344 jurisdictions; and tertiary medical service areas consist of 52 areas. The secondary medical area is a region where general inpatient medical care can be provided. In addition, the medical provision system is planned based on area unit to maintain medical resources (e.g., number of beds and number of clinic facilities). The characteristics of each region with respect to HLE by prefecture levels have been reported in multiple studies. However, these studies have focused on health promotion and care prevention measures. There is a lack of assessment in units of administrative districts that are directly linked to administrative activities.

The Japanese population has been rapidly aging because of decreasing birth rates and increasing LE. In fact, Japan has the highest proportion of older adults in the world [24]. In 1999, the population of those aged 65 years and older was 16.7% [25]. In 2019, older adults comprised 28.4% of the total population [26]. This figure is expected to increase to 35.3% by 2040, 37.7% by 2050, and 38.1% by 2060 [27]. Rapid aging has a substantial effect on disease structure, which is leading Japan to an advanced stage of epidemiological transitions [12,28]. Older adults experience a higher number of chronic diseases and multiple morbidities that require long-term care and increase healthcare expenditure. However, the characteristics of population aging and epidemiological transition differ by region, which can have a significant impact on healthcare system performance [29]. These factors influence the social determinants of health and may increase imbalances within the healthcare system [30]. Thus, it is important to develop a healthcare system that matches the actual conditions of each region. However, the actual distribution of the difference in HLE among secondary medical areas has yet to be clarified. Therefore, understanding the regional characteristics of the medical service system related to HLE in secondary medical areas is important to have a more effective approach in extending HLE and healthy aging policies.

In Japan, one of the most important policy challenges is the creation of an economically active aging society and a strong healthcare system to sustain it. Although the medical provision system in secondary medical areas is designed to be modified as necessary, it cannot always address regional disparities in healthcare resource distribution [31]. Japan is ranked 18th among OECD member states for low-cost medical care, and ranked first for its high level of health attainment and HLE [32,33]. Japan has one of the best healthcare systems in the world in terms of availability and cost. Many researchers attribute the success of its healthcare system to the dexterous balance of supply and demand and control over medical costs through the universal health insurance system [12,13]. However, Japan’s super-aging population is putting more pressure on its health system’s sustainability. Although the characteristics of the Japanese healthcare system have the advantages of healthcare availability, such healthcare systems cannot meet the needs of a super-aging society. Prior studies suggest that LE is influenced by healthcare resources, such as hospital bed capacity, healthcare workforce, and healthcare expenditure [34,35]. Healthcare resources have short- and long-term effects on health, which have been studied in terms of health output (e.g., number of medical doctors) or health outcomes (e.g., LE and mortality rate). However, the effects of healthcare resources on the average LE and HLE have not been fully elucidated. 

Ultimately, good overall population health is key to a productive and developed society. Healthcare resources, including number of beds and workforce size, is one of the most important factors that influence health status [36]. Although Japan’s healthcare system is the most organized in the world, there are functional differences between regions that need to be addressed [37]. There is a gap in terms of equal distribution of beds, staff, and doctors among all prefectures. Thus, it is necessary to clarify the relationship between medical care resources (i.e., hospital beds, healthcare workforce, home healthcare service, and healthcare expenditure) and HLE to contribute to developing effective policies to extend HLE.

### Current Study

This study aimed to identify the descriptive features of the distribution of HLE across secondary medical areas in Japan. In addition, it sought to clarify the relationship between healthcare resources, LE, and HLE through a geographical study of all secondary medical areas in Japan. The findings may have many implications for other countries with super-aging societies. However, research into the descriptive features of HLE and the associations between LE, HLE, and healthcare resources in the context of Japan is limited. This study comes at a time when health policymakers are reviewing and assessing priorities for action in Japan. It supports mapping of HLE in Japan, and the findings may contribute to the development of national and region-specific health policies for healthcare resources.

## 2. Materials and Methods

### 2.1. Measures

#### 2.1.1. Outcome Variables: Life Expectancy and Healthy Life Expectancy

We estimated HLE across all 344 secondary medical areas in Japan using the Sullivan method, which is based on age-specific death rates and years lived with disability [38]. In this method, by applying age-specific prevalence rates of a particular unhealthy state to a life table function (i.e., number of person-years lived in each age interval), the total LE is divided into person-years lived with good health (i.e., HLE). HLE is a measure of population health that estimates the expected number of “healthy years” (i.e., years spent in good health) of individuals at a given age. In addition, LE at birth was calculated using the population age standard.

To calculate LE and HLE, population data as of 2017 were obtained from the resident registry of Japan [39]. Mortality data were obtained from the Vital Statistics of Total Deaths in 2016–2018 [40]. Data on care needs were obtained from the Report on Long-Term Care Insurance Services. In this study, “healthy” was defined as the period of time spent without limitation in daily activities, while “unhealthy” was the period of time spent with limitations in daily activities. The Japanese care system is divided into care levels from 1 through 5, based on individuals’ care needs as certified by Japan’s long-term care insurance system (see Table S1) [41,42]. Data on unhealthy people, which included those at care level 2 (almost bedridden) and higher, were obtained from the 2017 long-term care insurance data [43]. The present study classified those at level 2 or greater as “having care needs” (i.e., unhealthy); all other levels were classified as “almost no care needs” (i.e., healthy).

#### 2.1.2. Explanatory Variable: Healthcare Resources

In this study, we used healthcare resources (i.e., hospital beds, healthcare workforce, home healthcare service, and healthcare expenditure) as the explanatory variable. The population ratio of healthcare resources across all 344 secondary medical areas was calculated [44,45,46]. We used data from the Survey of Medical Institutions, Annual Report on Health, Labour and Welfare, and National Database of Health Insurance Claims and Specific Health Checkups of Japan [44,45,46]. Hospital beds (i.e., number of beds) included curative (acute), rehabilitative, and long-term care beds per 1000 residents. The healthcare workforce (i.e., number of health professionals) consisted of doctors, nurses, and therapists per 1000 residents. Therapists included physical, occupational, and speech-hearing therapists. Home healthcare services (i.e., number of facilities) included support hospitals and clinics for home healthcare per 1000 residents, and home-visit care facilities per 1000 residents. The standard for healthcare facilities established by the Ministry of Health, Labour and Welfare requires hospitals and clinics to supplement home healthcare; medical facilities support patients living at home by providing medical care 24 hours a day [47]. A facility with at least 20 beds is considered a “support hospital of home health care”, while a facility with fewer than 20 beds is a “support clinic of home health care”.

#### 2.1.3. Confounding Variable: Population Density

Studies show that population density is significantly related to healthcare resources [37,48,49]. Considering that the location of healthcare resources is largely determined by the population of the area, we investigated the associations between healthcare resources, LE, and HLE at birth by performing partial correlation analyses after controlling for population density using data obtained from the Population Census of Japan [50]. Since the population density has a logarithmic normal distribution, the logarithm of the population density was calculated.

### 2.2. Statistical Analyses

IBM SPSS Statistics Version 26 (IBM Corp, Armonk, NY, USA) was used for data analyses. To examine the descriptive features of the distribution of HLE across secondary medical areas in Japan, it was divided into five categories by 20th percentiles and gender. Moreover, maps of HLE levels across Japan were drawn [51]. A chi-squared test was performed to assess the differences in HLE among eight regions (i.e., Hokkaido, Tohoku, Kanto, Chubu, Kinki, Chugoku, Shikoku, and Kyushu-Okinawa regions) by gender. 

Associations between healthcare resources, LE, and HLE at birth were assessed using partial correlation analyses controlling for population density. In the analysis, correlations were significant at the *p* < 0.001 level (two-tailed), and correlation coefficients greater than 0.2 were considered to indicate a positive correlation.

## 3. Results

### 3.1. Descriptive Statistics for Life Expectancy and Healthy Life Expectancy at Birth

Table 1 shows the descriptive statistics for LE and HLE at birth in years. The mean HLE at birth was 79.21 years (standard deviations 0.85) for males and 83.75 years (standard deviations 0.62) for females. Furthermore, a comparison of HLE in secondary medical areas revealed differences of 4.46 years (minimum 76.90, maximum 81.36) for males and 3.46 years (minimum 81.99, maximum 85.45) for females.

**Figure 1:**
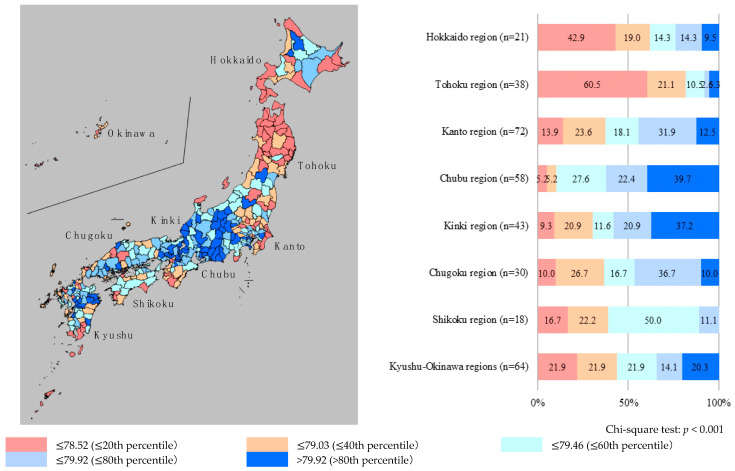
Distribution of healthy life expectancy for males in each secondary medical area. Note: Healthy life expectancy was divided into five categories, by 20th percentiles. The “n” means the number of secondary medical areas in eight regions (Hokkaido, Tohoku, Kanto, Chubu, Kinki, Chugoku, Shikoku, and Kyushu-Okinawa regions).

**Figure 2:**
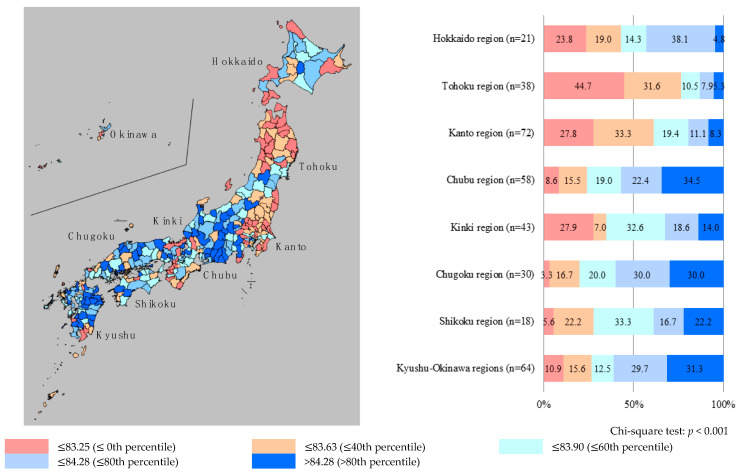
Distribution of healthy life expectancy for females in each secondary medical area. Note: Healthy life expectancy was divided into five categories, by 20th percentiles; The “n” means the number of secondary medical areas in eight regions (Hokkaido, Tohoku, Kanto, Chubu, Kinki, Chugoku, Shikoku, and Kyushu-Okinawa regions).

The nationwide distributions of HLE are presented in Figure 1 for males and Figure 2 for females. In the current study, chi-squared tests were performed to analyze the differences in HLE among eight regions (i.e., Hokkaido, Tohoku, Kanto, Chubu, Kinki, Chugoku, Shikoku, and Kyushu-Okinawa regions) by gender. For males, there were significant differences in HLE among the regions (*p* < 0.001). The proportion of secondary medical areas with short HLE tended to be higher in the northern part of Japan (Hokkaido and Tohoku regions), while the proportion of areas with high HLE was higher in the central (Chubu region) and west-central (Kinki regions) parts. Similarly, for females, there were significant differences in HLE among the regions (*p* < 0.001). The proportion of secondary medical areas with short HLE was higher in the northern (Tohoku region) and northern-central (Kanto region) parts of Japan, while the proportion of areas with high HLE was higher in the central (Chubu region) and southern (Kyushu-Okinawa regions) parts. These results indicated significant regional disparities; the common point is that for both males and females, the proportion of areas with short HLE was high in the northern part of Japan (Tohoku region), while the proportion of areas with high HLE was high in the central part (Chubu region).

### 3.2. Associations Between Healthcare Resources, Life Expectancy, and Healthy Life Expectancy at Birth

Data for healthcare resource variables (i.e., hospital beds, healthcare workforce, community healthcare service, and healthcare expenditure) are shown in Table 2. Associations between healthcare resources, LE, and HLE at birth are shown in Table 3 and Table 4. For both males and females, the numbers of hospital beds were not correlated with LE and HLE, and no significant correlation was observed according to the criteria of this study (i.e., correlations were significant at the *p <* 0.001 level, and correlation coefficients greater than 0.2 were considered to indicate a positive correlation). However, for males, the number of curative (acute) care beds per 1000 residents tended to correlate negatively with LE (*r* = −0.122, *p* = 0.023) and HLE (*r* = −0.129, *p* = 0.017). In contrast, for females, the number of rehabilitative care beds per 1000 residents tended to correlate positively with LE (*r* = 0.126, *p* = 0.019) and HLE (*r* = 0.157, *p* = 0.003), and long-term care beds per 1000 residents tended to correlate positively with HLE (*r* = 0.117, *p* = 0.030).

Regarding the healthcare workforce, there was no significant positive correlation with LE and HLE for males; however, therapists per 1000 residents tended to be positively correlated with HLE (*r* = 0.115, *p* = 0.033). For females, doctors per 1000 residents were significantly positively correlated with LE (*r* = 0.220, *p* < 0.001), and therapists per 1000 residents were significantly positively correlated with HLE (*r* = 0.242, *p* < 0.001). Meanwhile, doctors per 1000 residents tended to correlate positively with HLE (*r* = 0.154, *p* = 0.004), nurses per 1000 residents tended to correlate positively with LE (*r* = 0.119, *p* = 0.027) and HLE (*r* = 0.134, *p* = 0.013), and therapists per 1000 residents tended to correlate positively with LE (*r* = 0.198, *p* < 0.001).

In terms of home healthcare service, there were no significant correlations with LE and HLE for males. However, support hospitals for home healthcare per 1000 residents tended to correlate positively with HLE (*r* = 0.106, *p* = 0.049). Moreover, support clinics for home healthcare and home-visit care facilities per 1000 residents tended to correlate positively with LE (*r* = 0.155, *p* = 0.004 and *r* = 0.145, *p* = 0.007, respectively) and HLE (*r* = 0.154, *p* = 0.004 and *r* = 0.134, *p* = 0.013, respectively). For females, support clinics for home healthcare and home-visit care facilities per 1000 residents were significantly positively correlated with both LE (*r* = 0.226, *p* < 0.001 and *r* = 0.268, *p* < 0.001, respectively) and HLE (*r* = 0.222, *p* < 0.001 and *r* = 0.231, *p* < 0.001, respectively). Support hospitals for home healthcare per 1000 residents tended to correlate positively with LE (*r* = 0.182, *p* < 0.001) and HLE (*r* = 0.190, *p* < 0.001).

For healthcare expenditure, for males, dentistry expenditure per capita was significantly positively correlated with both LE (*r* = 0.228, *p* < 0.001) and HLE (*r* = 0.237, *p* < 0.001), while outpatient expenditure per capita tended to correlate positively with LE (*r* = 0.180, *p* < 0.001) and HLE (*r* = 0.176, p = 0.001). For females, healthcare expenditure did not have a significant correlation with LE and HLE. However, hospitalization, outpatient, and dentistry expenditure per capita tended to correlate positively with LE (*r* = 0.192, *p* < 0.001, *r* = 0.173, *p* = 0.001, and *r* = 0.132, *p* = 0.015, respectively) and HLE (*r* = 0.180, *p* < 0.001, *r* = 0.141, *p* = 0.009, and *r* = 0.131, *p* = 0.015, respectively).

## 4. Discussion

This study aimed to reveal the descriptive features of the distribution of HLE across secondary medical areas in Japan. In addition, the relationship among healthcare resources, LE, and HLE was examined through a geographical study of all secondary medical areas in Japan. The findings revealed significant regional disparities regarding HLE despite controlling for population density. This indicates the need for action and execution of programs aimed at reducing regional disparities. For males, the proportion of secondary medical areas with short HLE was higher in the northern part of Japan (Hokkaido and Tohoku regions), while areas with higher rates of high HLE were in the central (Chubu region) and west-central (Kinki regions) parts. For females, the proportion of secondary medical areas with short HLE was higher in the northern (Tohoku region) and northern-central (Kanto region) parts of Japan, while areas with higher rates of high HLE were in the central (Chubu region) and southern (Kyushu-Okinawa regions) parts. The above results indicate that for both males and females, the proportion of areas with short HLE was higher in the northern part of Japan (Tohoku region), and the areas with higher rates of high HLE were in the central part (Chubu region). Thus, this suggests that there are inequalities in HLE across secondary medical areas in Japan.

Previous research suggests that healthcare resources, including the number of beds, can affect health. However, in this study, hospital beds were not significantly correlated with LE and HLE for either males or females at birth. This lack of correlation might be explained by the fact that hospital beds are well-deployed in Japan [52,53]. Although there was no significant association between the number of beds and LE and HLE, a negative trend for acute care beds and health outcomes was observed; that is, for males, curative (acute) care beds per 1000 residents were negatively correlated with LE and HLE. This finding is consistent with previous studies that found that providing an excessive number of beds was negatively associated with health outcomes [34]. Overall, the level of health resources, including human and physical resources, is positively correlated with better health outcomes (i.e., decreased death rates and prolonged life). Ultimately, policymakers should consider the optimal levels of health resources in terms of number of physicians, nurses, and beds to achieve better health outcomes. In addition, they should consider the potential negative effects of oversupply on population health. 

Regarding the healthcare workforce, for females, doctors per 1000 residents were significantly positively correlated with LE and tended to correlate positively with HLE; conversely, therapists per 1000 residents were significantly positively correlated with HLE and tended to correlate positively with LE. For males, therapists per 1000 residents tended to correlate positively with HLE. Prior studies suggested that healthcare resources have short- and long-term effects on health. In addition, multiple studies revealed that countries with higher levels of human health resources (e.g., number of physicians) typically have better overall population health (e.g., LE and mortality rate) [54]. Studies on international differences on the impact of the number of doctors on health reported that doctors per capita were an important determinant of mortality [55,56,57]. Mortality rate has a negative correlation with the number of doctors, which seems reasonable since a sufficient number of doctors who can diagnose diseases and provide appropriate care services can mitigate certain causes of death. Therefore, the number of doctors may be related to LE and HLE. 

Furthermore, in this study, the number of therapists was also significantly associated with HLE; Rehabilitative health services are expected to extend HLE, since rehabilitation is aimed at optimizing individual functions related to health status [58]. Rehabilitation practitioners include physical therapists, occupational therapists, and speech-hearing therapists. As aging of the population has a serious impact on social systems, it is important to address people’s functioning, including restricted functions that affect their daily activities. Considering the effect of healthcare worker availability on health, the need to improve cross-region heterogeneity is significant. Thus, the healthcare workforce can be a facilitating factor of LE and HLE. However, the link between rehabilitation of human resources and health outcomes has not been fully validated and further evaluations are required in the future.

Home health resources were related to LE and HLE. For females, support clinics for home healthcare and home-visit care facilities per 1000 residents had significant positive correlations with both LE and HLE. On the other hand, for males, support clinics for home healthcare and home-visit care facilities per 1000 residents tended to be positively correlated with LE and HLE. Visiting medical care can be improved by increasing the efficiency and collaboration between care service dealers, clinics, and hospitals. Improving home-based medical care and nursing, which include end-of-life care that is in keeping with the individual’s values, can enhance both LE and HLE. Studies have noted that access to a primary care service has a strong and significant influence on longer LE and lower mortality rates [59,60]. Primary care typically involves a wide range of contents, including prevention and treatment. Prior studies at the regional level demonstrated that higher availability of primary care services was associated with fewer incidences of disabilities and mortality [59,60]. Although the Japanese healthcare system is open and flexible regarding healthcare provision and access [11], it cannot be said that home-based medical care is adequate, as has been noted in this study. Moreover, ranges of distribution in home healthcare service among secondary medical areas were observed in this study (see Table 2). Home healthcare services can be a facilitating factor of LE and HLE. However, while the effect of hospital healthcare resources on health outcomes has been reported, the link between home healthcare services and health outcomes has not been fully clarified, thus warranting further investigation in the future.

Regarding healthcare expenditure, for males, dentistry expenditure per capita was significantly positively correlated with both LE and HLE; for females, dentistry expenditure per capita tended to correlate positively with both LE and HLE. Dental expenditure seems to be associated with the level of access to oral care. To prevent loss of teeth, it is important to maintain oral health [61,62]. Although the basic step to maintaining oral health is oral self-care, making an accurate self-assessment of oral health status is difficult. Thus, it is important for older adults to visit dental health experts for regular prevention and treatment. Oral health is one of the key markers of overall health, well-being, and QOL [63]. In addition, oral diseases are risk factors for various diseases [64,65,66]. For instance, oral diseases are related to physical illnesses, such as infections and cardiovascular diseases. In addition, missing teeth is a risk factor for decline of cognitive competence [67]. Oral diseases are chronic and cumulative in nature and aggravate over time [68]. In addition, dental care costs may be affected by socioeconomic factors; socioeconomic differences are associated with LE and healthy and disease-free LE [69]. These findings concur with the present findings, which highlights the importance of providing appropriate prevention and treatment services to maintain dental health and functioning in later life.

In this study, regarding the association between healthcare resources and health outcomes, males had lower or non-significant correlation values compared to females. Lower point estimates among males compared to females can be explained by the fact that the difference between LE and HLE (i.e., unhealthy life expectancy) is higher among females than among males; in this study, the differences were 1.41 years and 3.12 years for males and females, respectively. In addition, similar to Japan, unhealthy life expectancy tends to be higher in females than in males in other countries [10]. In general, males and females differ in terms of various health outcomes, including mortality and morbidity [70,71]. In the present study, a shorter LE and HLE among males compared to females, as well as other limitations, may have affected the relationship between LE, HLE, and healthcare resources. Factors related to lifestyle that affect morbidity and mortality, including health-related behavior, might explain the gender gap in health expectancy [72,73,74,75,76,77,78]. However, the predictors of gender differences in environmental factors and HLE have not been fully clarified. In order to continue developing effective approaches, it is necessary to identify the factors affecting gender differences.

In this study, the distribution of HLE conducted at the secondary care area level is consistent with a previous study conducted at the prefecture level in Japan [21]. The study conducted at the prefecture level suggested that behavioral risk factors made a greater contribution to both disability-adjusted life years and mortality, and that the most important behavioral risk factors for health outcomes were unhealthy diet and smoking. Substantial opportunities for a healthier population exist via modifiable risk factor approaches. However, since it is difficult to identify a specific factor that affects HLE, there are possibilities of interplay among various factors, including healthcare system and lifestyle [14,15]. In this study, there were no strong associations between healthcare resources and HLE; however, factors other than healthcare resources could have a possible impact on HLE. People living in regions with higher HLE might have better lifestyles, including more efficient prevention and treatment of diseases. Thus, to continue developing effective approaches, it is necessary to identify the factors from multifaceted viewpoints. It is important to consider not only the optimal levels of health resources, but also lifestyle factors, in order to improve HLE.

### Limitations

This study had several limitations. First, there is a possible bias of geographical fallacy, as this study model was a geographical study. Second, although we analyzed the relationship between medical resources, LE, and HLE, adjusting for population density alone, other factors were not adjusted; thus, it is difficult to determine a specific factor. Various other confounding factors may need to be controlled for in future studies. Third, although the findings of this study concur with some of those of previous studies conducted in other countries, it should be noted that this study was conducted only in Japan. As all data were collected in Japan, it is difficult to generalize the results to other countries. 

## 5. Conclusions

In the present study, inequalities in HLE were found despite controlling for population density. This indicates the need to promote social policies designed to reduce regional disparities. As described above, some factors related to healthcare resources were significantly related to LE and HLE; the numbers of doctors and therapists, support clinics for home healthcare facilities and home-visit treatments, and dentistry expenditure per capita were positively correlated with LE and HLE. Most of our findings were consistent with previous studies conducted in other countries that have different levels of healthcare systems; multiple studies have shown that the most important factor that contributes to a population’s health status is healthcare resources, which include the number of beds and healthcare professionals. On the other hand, this study may indicate an important factor in countries with high-quality healthcare systems, since there is limited research on the relationship between healthcare resources, such as healthcare workforce, home healthcare facilities, and home-visit treatments, and HLE. Ultimately, policymakers should consider the optimal levels of healthcare resources in order to improve LE and HLE. Our results suggest the necessity to consider the geographical redistribution of healthcare resources. In addition, further studies are needed to ensure a more effective and balanced geographical distribution of healthcare systems in Japan.

## Figures and Tables

**Table 1:** Descriptive statistics for life expectancy and healthy life expectancy (years) at birth.

Variables	Males				Females			
	M	SD	Min	Max	M	SD	Min	Max
Life expectancy	80.62	0.87	78.30	82.79	86.87	0.62	85.05	88.36
Healthy life expectancy	79.21	0.85	76.90	81.36	83.75	0.62	81.99	85.45

Note: Life expectancy and healthy life expectancy were calculated by the Sullivan method; M: Mean; SD: Standard deviation; Min: Minimum value; Max: Maximum value.

**Table 2:** Descriptive statistics of healthcare resource variables.

Variables	M	SD	Min	Max
Hospital beds (number of beds per 1000 residents)				
Curative (acute) care	6.06	1.77	1.57	14.19
Rehabilitative care	1.41	0.83	0.00	5.05
Long-term care	3.45	2.31	0.00	17.33
Healthcare workforce (number of healthcare providers per 1000 residents)				
Doctors	2.45	0.87	1.12	11.89
Nurses	9.55	2.87	3.95	19.61
Therapists	1.16	0.57	0.08	4.30
Home healthcare service (number of facilities per 1000 residents)				
Support hospital of home healthcare	0.01	0.01	0.00	0.10
Support clinic of home healthcare	0.10	0.06	0.00	0.35
Home-visit care facility	0.20	0.08	0.06	0.47
Healthcare expenditure (JPY per capita)				
Hospitalization	153,129.53	31,146.30	91,904.53	253,059.03
Outpatient	195,347.25	17,342.34	135,962.50	276,722.07
Dentistry	23,966.78	2887.87	14,294.20	32,158.29

Note: The facilities with at least 20 beds are “support hospital of home health care”, while the facilities with fewer than 20 beds are “support clinic of home health care”. Therapists include physical therapists, occupational therapists, and speech-hearing therapists. JPY: Japanese yen, M: Mean, SD: Standard deviation; Min: Minimum value; Max: Maximum value.

**Table 3:** Associations between healthcare resources, life expectancy, and healthy life expectancy at birth for males.

Variables	Life Expectancy			Healthy Life Expectancy		
	*r*	*p* Value	df	*r*	*p* Value	df
Hospital beds (number of beds per 1000 residents)						
Curative (acute) care	−0.122	0.023	341	−0.129	0.017	341
Rehabilitative care	0.022	0.686	341	0.034	0.533	341
Long-term care	−0.010	0.860	341	0.014	0.801	341
Healthcare workforce (number of healthcare providers per 1000 residents)						
Doctors	0.076	0.163	341	0.046	0.395	341
Nurses	−0.046	0.396	341	−0.044	0.421	341
Therapists	0.099	0.067	341	0.115	0.033	341
Home healthcare service (number of facilities per 1000 residents)						
Support hospital of home healthcare	0.106	0.051	341	0.106	0.049	341
Support clinic of home healthcare	0.155	0.004	341	0.154	0.004	341
Home-visit care facility	0.145	0.007	341	0.134	0.013	341
Healthcare expenditure (JPY per capita)						
Hospitalization	0.002	0.964	341	0.004	0.940	341
Outpatient	0.180	<0.001	341	0.176	0.001	341
Dentistry	0.228 *	<0.001	341	0.237 *	<0.001	341

Note: Partial correlation analyses were performed by controlling for population density; * Correlations were significant at the *p <* 0.001 level (two-tailed), and correlation coefficients greater than 0.2 were considered to indicate a positive correlation.

**Table 4:** Associations between healthcare resources, life expectancy, and healthy life expectancy at birth for females.

Variables	Life Expectancy			Healthy Life Expectancy		
	*r*	*p* Value	df	*r*	*p* Value	df
Hospital beds (number of beds per 1000 residents)						
Curative (acute) care	0.040	0.460	341	0.018	0.746	341
Rehabilitative care	0.126	0.019	341	0.157	0.003	341
Long-term care	0.068	0.208	341	0.117	0.030	341
Healthcare workforce (number of healthcare providers per 1000 residents)						
Doctors	0.220 *	<0.001	341	0.154	0.004	341
Nurses	0.119	0.027	341	0.134	0.013	341
Therapists	0.198	<0.001	341	0.242 *	<0.001	341
Home healthcare service (number of facilities per 1000 residents)						
Support hospital of home healthcare	0.182	<0.001	341	0.190	<0.001	341
Support clinic of home healthcare	0.226 *	<0.001	341	0.222 *	<0.001	341
Home-visit care facility	0.268 *	<0.001	341	0.231 *	<0.001	341
Healthcare expenditure (JPY per capita)						
Hospitalization	0.192	<0.001	341	0.180	<0.001	341
Outpatient	0.173	0.001	341	0.141	0.009	341
Dentistry	0.132	0.015	341	0.131	0.015	341

Note: Partial correlation analyses were performed by controlling for population density; * Correlations were significant at the *p <* 0.001 level (two-tailed), and correlation coefficients greater than 0.2 were considered to indicate a positive correlation.

## Supplementary Materials

The following are available online at https://www.mdpi.com/1660-4601/17/17/6301/s1, Table S1: Types of long-term care (Japanese care system).
