# Erratum: Hosokawa, R., et al. Associations between Healthcare Resources and Healthy Life Expectancy: A Descriptive Study across Secondary Medical Areas in Japan. *Int. J. Environ. Res. Public Health* 2020, *17*, 6301

**DOI:** 10.3390/ijerph17207521

**Published:** 2020-10-16

**Authors:** Rikuya Hosokawa, Toshiyuki Ojima, Tomoya Myojin, Jun Aida, Katsunori Kondo, Naoki Kondo

**Affiliations:** 1 Department of Human Health Sciences, Graduate School of Medicine, Kyoto University, Kyoto 606-8507, Japan; 2 Department of Community Health and Preventive Medicine, Hamamatsu University School of Medicine, Shizuoka 431-3192, Japan; ojima@hama-med.ac.jp; 3 Department of Public Health, Health Management and Policy, Nara Medical University, Nara 634-8521, Japan; motoya1014@gmail.com; 4 Department of Oral Health Promotion, Graduate School of Medical and Dental Sciences, Tokyo Medical and Dental University, Tokyo 113-8549, Japan; junaida916@gmail.com; 5 Division for Regional Community Development, Liaison Center for Innovative Dentistry, Graduate School of Dentistry, Tohoku University, Miyagi 980-8575, Japan; 6 Center for Preventive Medical Sciences, Chiba University, Chiba 263-8522, Japan; kkondo@kkondo.net; 7 Center for Well-being and Society, Nihon Fukushi University, Aichi 470-3295, Japan; 8 Center for Gerontology and Social Science, National Center for Geriatrics and Gerontology, Aichi 474-8511, Japan; 9 Department of Health and Social Behavior, School of Public Health, The University of Tokyo, Tokyo 113-0033, Japan; nkondo@m.u-tokyo.ac.jp; 10 Department of Health Education and Health Sociology, School of Public Health, The University of Tokyo, Tokyo 113-0033, Japan

Due to an error during production, the legend of Figure 2 in the published paper [1] was incorrect.

## Incorrect:

≤0th percentile

**Figure:**
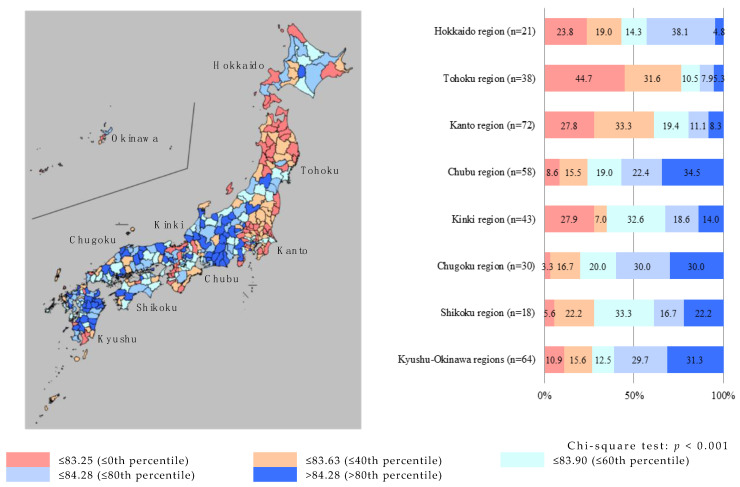


## Correct:

≤20th percentile

**Figure:**
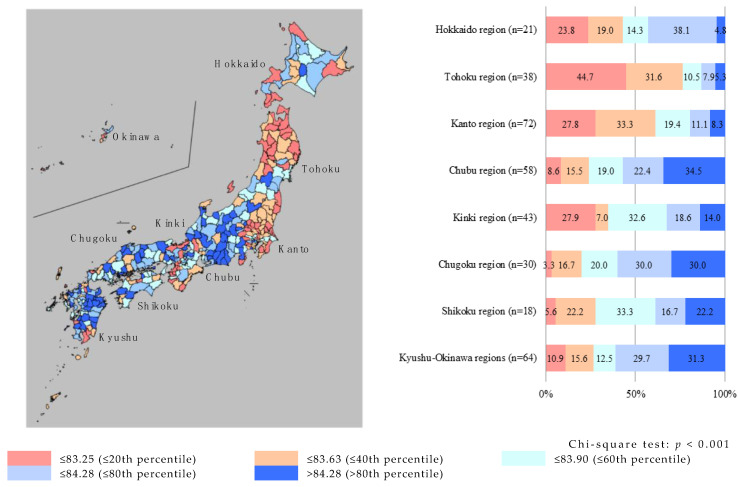


We apologize for any inconvenience caused to readers or authors by these changes. The article will be updated and the original will remain available on the article webpage.
