# Supplementary material for: Associations between Healthcare Resources and Healthy Life Expectancy: A Descriptive Study across Secondary Medical Areas in Japan
Source: Int J Environ Res Public Health. 2020 Aug 29;17(17):6301. doi: 10.3390/ijerph17176301 (PMC7503367; doi:10.3390/ijerph17176301)
Supplement: Supplementary file 1 [file ijerph-17-06301-s001.pdf]

Table S1. Types of long-term care (Japanese care system).

Types of Long-Term Care	Indicative Conditions
Care Level 1	People face difficulty in performing essential daily life activities by themselves.
Care Level 2	People are in a state similar to that detailed under Care Level 1, but require more care to be able to perform essential daily life activities.
Care Level 3	Compared with the state of those in Care Level 2, people’s ability to perform essential daily life and task- based activities are significantly lower. As a result, they require almost constant care.
Care Level 4	People are in a state similar to that detailed under Care Level 3, but their ability to act is lower. As a result, they face difficulty living without constant care.
Care Level 5	People’s ability to act is even lower than that of individuals in Care Level 4 category. As a result, they require almost constant care to live.
